# A giant subcutaneous leiomyosarcoma arising in the inguinal region

**DOI:** 10.1186/1477-7819-3-14

**Published:** 2005-02-24

**Authors:** Kazuhito Yajima, Yoshio Shirai, Nobuhiro Fujita, Daisuke Sato, Hajime Umezu, Katsuyoshi Hatakeyama

**Affiliations:** 1 Division of Digestive and General Surgery, Niigata University Graduate School of Medical and Dental Sciences, 1-757 Asahimachi-dori, Niigata City, 951-8510 Japan; 2 Division of Pathology, Niigata University Medical and Dental Hospital, 1-754 Asahimachi-dori, Niigata City, 951-8520 Japan

## Abstract

**Background:**

Subcutaneous leiomyosarcoma is a rare condition that accounts for 1% to 2% of all superficial soft tissue malignancies. Approximately 10% of cases arise in the trunk, although the extremities are the most commonly affected.

**Case presentation:**

We report herein the case of a 31-year-old man with a subcutaneous leiomyosarcoma, measuring 124 × 105 mm, arising in the left inguinal region. A wide local excision (with a resection margin ≥ 20 mm) was performed. Histological examination of the resected specimen revealed a leiomyosarcoma with high cellularity and two mitoses per 10 high-power fields. The patient remains well with no evidence of disease 5 years and 8 months after the operation.

**Conclusion:**

This is the first reported case of subcutaneous leiomyosarcoma arising in the inguinal region and also one of the largest tumors reported. The experience of this case and a review of the English-language literature (PubMed, National Library of Medicine, Bethesda, MD, USA) suggest that a resection margin of ≥ 10 mm is recommended when excising this rare tumor.

## Background

Subcutaneous leiomyosarcoma arises from smooth muscle in the walls of arterioles and veins. It is a rare tumor accounting for 1% to 2% of all superficial soft tissue malignancies [1-4]. It usually occurs in patients between 50 and 70 years of age [1,2,5], with a male predominance ranging from 2:1 to 3:1 [2,4]. Although most tumors present as a subcutaneous nodule in the extremities, usually measuring 30 mm or less in diameter, about 10% of cases arise in the trunk [1,2,4]. We herein report the case of a patient with a giant subcutaneous leiomyosarcoma arising in the inguinal region.

## Case presentation

**Figure 1:**
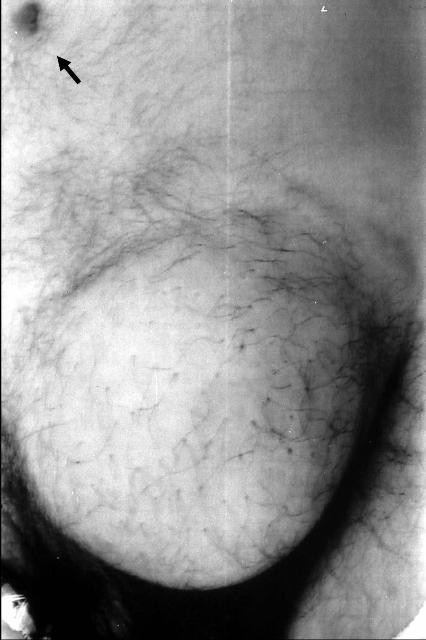
A fist-sized tumor arising in the subcutaneous adipose tissue in the inguinal region. An arrow indicates the navel.

**Figure 2:**
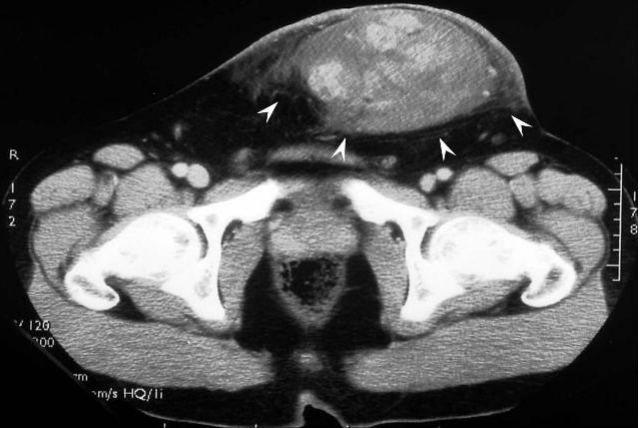
Computed tomography depicted a solid tumor with heterogeneous contrast enhancement (arrowheads) in the adipose tissue.

A 31-year-old man presented with a painless left inguinal tumor, which had gradually grown during the past six months. Physical examination on admission revealed a fist-sized subcutaneous tumor in the inguinal region. The overlying skin appeared normal without ulceration (Figure 1), and there was no inguinal lymphadenopathy. Computed tomography depicted a solid tumor with heterogeneous contrast enhancement in the adipose tissue, and no metastases to the liver and lung (Figure 2). With a tentative diagnosis of soft tissue sarcoma of unknown origin, a wide local excision (with a resection margin ≥ 20 mm) was performed.

**Figure 3:**
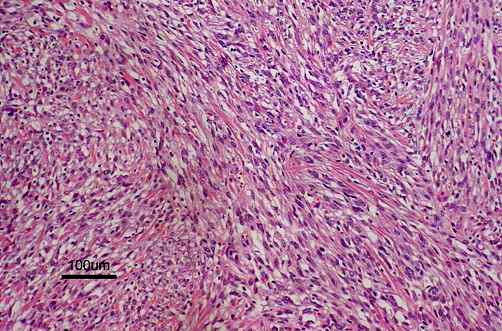
The tumor comprised spindle-shaped cells with high cellularity in parts (hematoxylin-and-eosin; original magnification, × 100).

**Figure 4:**
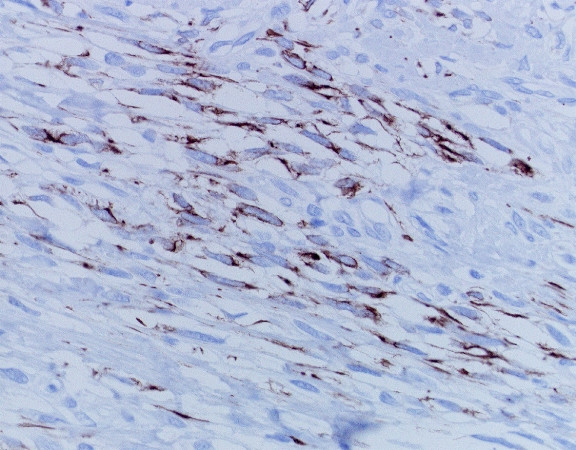
Spindle-shaped cells showing strong immunoreactivity for desmin in the cytoplasm (Desmin immunohistochemistry; original magnification, × 400).

The resected tumor, measuring 124 × 105 mm, was solid, encapsulated, and a homogeneous yellowish white in color, without central necrosis and hemorrhage on its cut surface. Routine histological examination with hematoxylin-and-eosin revealed that the tumor comprised spindle-shaped cells with high cellularity in parts (Figure 3) and two mitoses per 10 high-power fields. Immunohistochemistry with mouse monoclonal antibodies against desmin (D33, Dako Cytomation Japan Co. Ltd., Kyoto, Japan), alpha-smooth muscle actin (1A4, Dako Cytomation Japan Co. Ltd., Kyoto, Japan), vimentin (V9, Dako Cytomation Japan Co. Ltd., Kyoto, Japan) and S-100 protein (2A10, IBL-Japan Co. Ltd., Takasaki, Japan) was performed. As the tumor cells showed only immunoreactivity for desmin (Figure 4) and alpha-smooth muscle actin, a diagnosis of leiomyosarcoma of subcutaneous adipose tissue origin was confirmed.

The patient had an uneventful recovery and was discharged on the 9th postoperative day. As the resection margin was negative, no adjuvant treatment was given. He remains well with no evidence of disease 5 years and 8 months after excision.

## Discussion

Although subcutaneous leiomyosarcoma commonly arises in the lower extremities, it occasionally affects the trunk [1,2,4]. A review of the English-language literature (PubMed, National Library of Medicine, Bethesda, MD, USA) suggests that the case reported here is the first one arising in the inguinal region and involves one of the largest tumors reported thus far [2,7]. Earlier authors proposed several prognostic factors for soft tissue sarcomas (including subcutaneous leiomyosarcoma) [2,6-8]. Factors adversely affecting the prognosis include high mitotic index (≥ 5 mitoses per 10 high-power fields) [2], high histologic grade [6], extensive necrosis [7], nodular growth pattern [7], deep tumor [8], and large tumor size [8]. Among them, tumor size (≥ 5 cm) is the strongest independent prognostic factor [8]. In the current case, low mitotic index, low histologic grade and the absence of necrosis favored a good prognosis, while large tumor size, deep tumor and nodular growth pattern were adverse prognostic factors.

As subcutaneous leiomyosarcoma is resistant to radiotherapy and chemotherapy [2,3,9], surgical excision provides the only chance of cure. Prognosis after excision is generally considered poor, and even after a wide excision, local recurrence may occur in 40% to 60% of patients, followed by distant metastases in 20% to 40% of cases [1,2]. Contaminated margins contribute to the frequency of local recurrences [5,10-12]. In the current case, the resection margin of ≥ 20 mm successfully controlled the tumor. McKee *et al*., [12] demonstrated that resection margins ≥ 10 mm decreased the risk of both local and distant recurrences in patients with soft tissue sarcomas (including subcutaneous leiomyosarcoma). A resection margin of ≥ 10 mm is therefore recommended when excising this rare tumor.

## Competing interests

The author(s) declare that they have no competing interests.

## Authors' contributions

**KY**, **YS**, **NF**, and **DS **took part in the operation, performed the literature search and drafted the manuscript for submission. **HU **performed histological examination. **KH **supervised the preparation of the manuscript and edited the final version for publication. All authors read and approved the final manuscript.
